# Supplementary material for: Distilling nanoscale heterogeneity of amorphous silicon using tip-enhanced Raman spectroscopy (TERS) via multiresolution manifold learning
Source: Nat Commun. 2021 Jan 25;12:578. doi: 10.1038/s41467-020-20691-2 (PMC7835247; doi:10.1038/s41467-020-20691-2)
Supplement: Supplementary file 1 — Supplementary Information [file 41467_2020_20691_MOESM1_ESM.pdf]

Supporting information

Title

Distilling Nanoscale Heterogeneity of Amorphous Silicon using Tip-enhanced Raman Spectroscopy (TERS) via Multiresolution Manifold Learning

Guang Yang[1]*, Xin Li[1]*, Yongqiang Cheng[1], Mingchao Wang[2], Dong Ma[1], Alexei P. Sokolov [1,3], Sergei V. Kalinin[1] , Gabriel M.Veith[1] and Jagjit Nanda[1]*

1 Oak Ridge National Laboratory, Oak Ridge, TN 37831 USA

2 Department of Materials Science and Engineering, Monash University, Clayton, VIC 3800, Australia.

3 Department of Applied Physics and Materials, Tulane University, LA USA

4 Department of Chemistry, University of Tennessee, Knoxville, TN 37996 USA

*Corresponding author emails:
yangg@ornl.gov; lix3@ornl.gov; nandaj@ornl.gov

This manuscript has been authored by UT-Battelle, LLC under Contract No. DE-AC05-00OR22725 with the U.S. Department of Energy. The United States Government retains and the publisher, by accepting the article for publication, acknowledges that the United States Government retains a non-exclusive, paid-up, irrevocable, world-wide license to publish or reproduce the published form of this manuscript, or allow others to do so, for United States Government purposes. The Department of Energy will provide public access to these results of federally sponsored research in accordance with the DOE Public Access Plan (http://energy.gov/downloads/doe-public-access-plan).

Finite difference time domain (FDTD) simulation

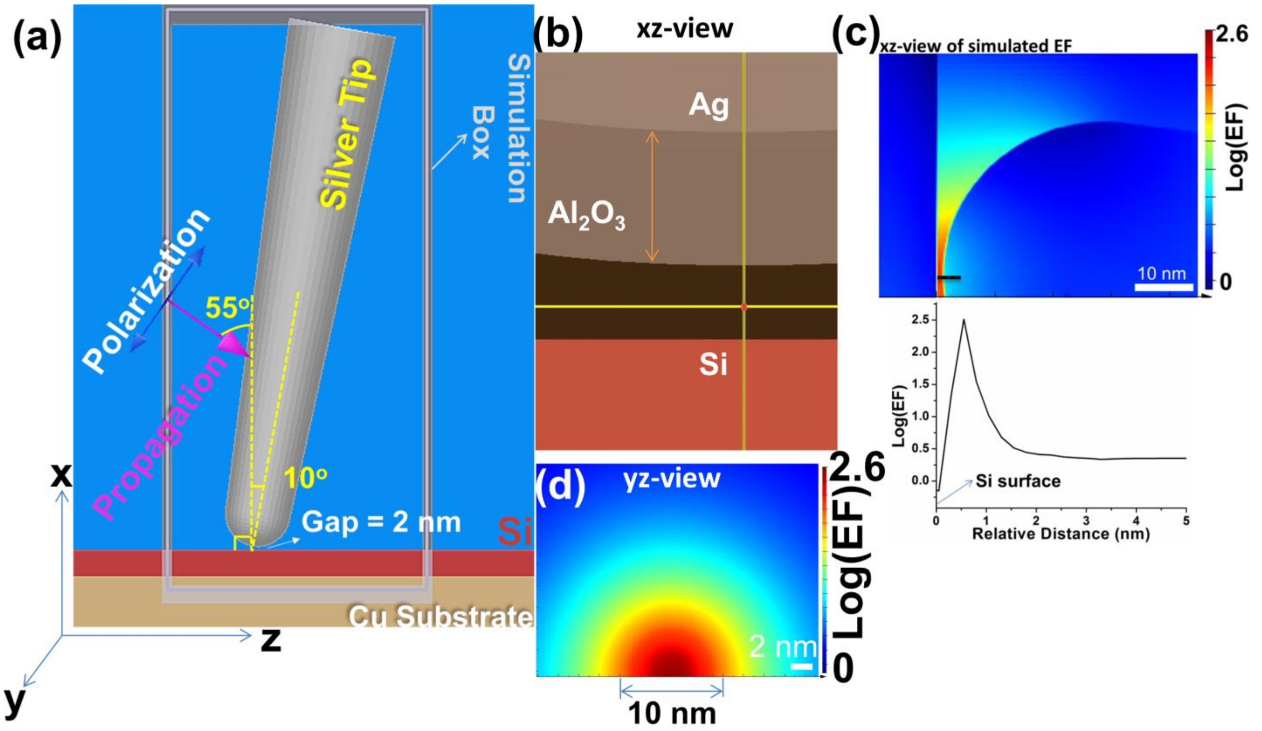

Figure S1. (a) Scheme of the FDTD model setup. (b) xz-plane view on the FDTD model at the tip-Si gap region (c) Simulated enhancement factor (EF) distribution around the tip apex. The line plot below presents the EF distribution in log scale along x-axis, with the origin at the Si surface. The log(EF) profile was generated along the black line cut. (d) Simulated enhancement factor (EF) distribution on the yz-plane 0.5 nm away from the Si surface.

Composite TERS mapping of various TERS modes on a-Si

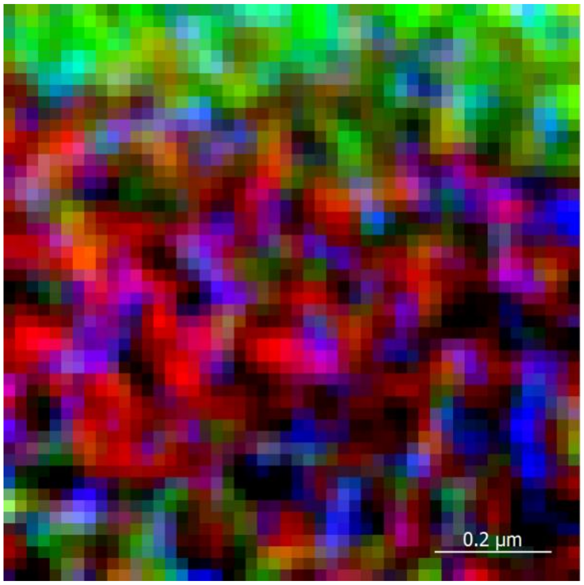

Figure S2. The composite TERS mapping combined ones shown in Figure 2(b-d).

Raman spectral comparison among different samples and values with respect to laser power

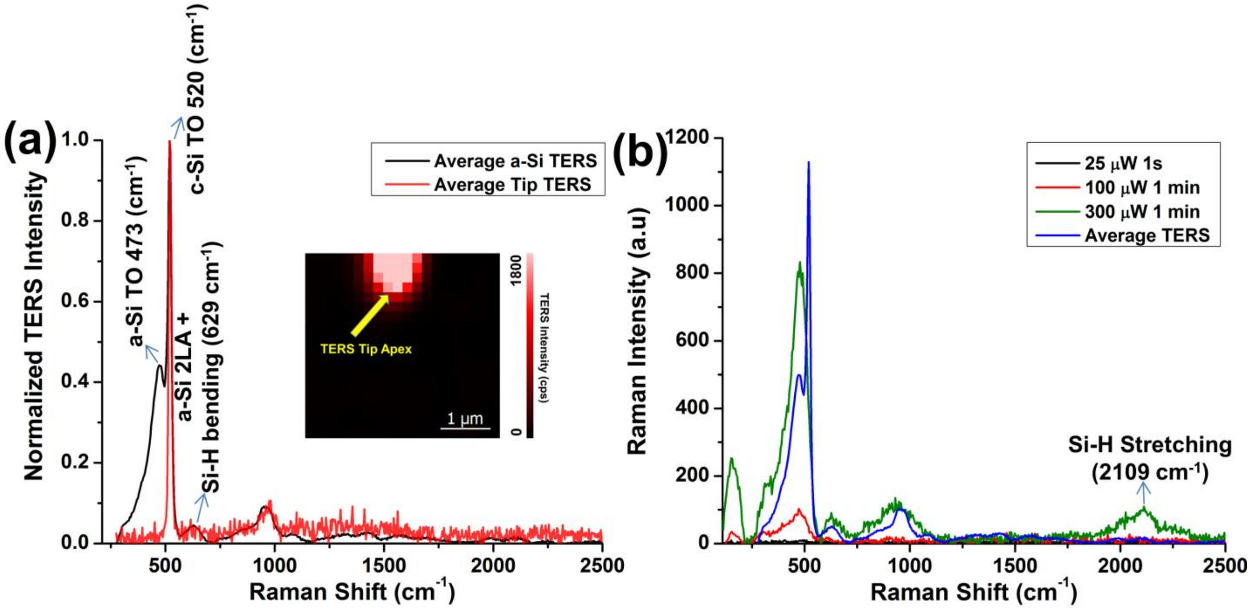

Figure S3. (a) Comparison between the normalized TERS spectra collected from the a-Si sample surface, and from the tip when it is far from (2 mm) the a-Si surface. The a-Si TERS spectrum was an average one over 2500 TERS spectra. The tip TERS spectra were averaged over 10 pixels from the TERS mapping of the tip shown in the inset. (b) Comparison among different micro-Raman spectra obtained using different values of laser power and collection time. The average TERS spectrum from the a-Si was used as a reference.

The TERS spectra collected from the a-Si surface composed of vibrational contributions from both of the amorphous silicon (a-Si) and crystal silicon (c-Si). The most distinguished band for the c-Si locates at 520 cm^{-1}, representing the first order TO mode. Its counterpart of a-Si locates at 473 cm^{-1}. A close inspection on the a-Si sample using a higher laser power at > 100 µW indicates the a-Si sample used in this study is in completely amorphous phase as no c-Si TO mode presents (Figure S3). It is worth emphasizing again that using the same experimental condition for TERS (i.e. laser power of 25 µW and collection time of 1s), the spectrum collected from a-Si with micro-Raman is almost featureless as shown in Figure S3(b). Further increase in the laser power and collection time leads to meaningful Raman spectrum of a-Si. With 100 µW laser power and 1 min collection time, no Si-H stretching mode at 2109 cm^{-1} was observed. The Si-H bending mode as reflected by the INS spectrum in Figure 6 may also show in the Raman or TERS spectrum at 629 cm^{-1}. But it possibly overlaps with the second order LA mode. The average TERS spectrum over 2500 spectra collected from the scanning area of a-Si does not reflect the Si-H stretching mode at 2109 cm^{-1}. This may be due to the fact that the total abundance of the Si-H group on a-Si surface is not high enough, and the Raman cross section of the Si-H stretching mode is low in comparison with other vibrational modes. Further increase the laser power to 300 µW with 1 min collection time, the Si-H stretching mode at 2109 cm^{-1} shows up. The exploration of the Si-H mode on a-Si surface using TERS necessitates an increased laser power and collection time with respect to the current conditions in the TERS setup.

The "tip-in and tip-out" test

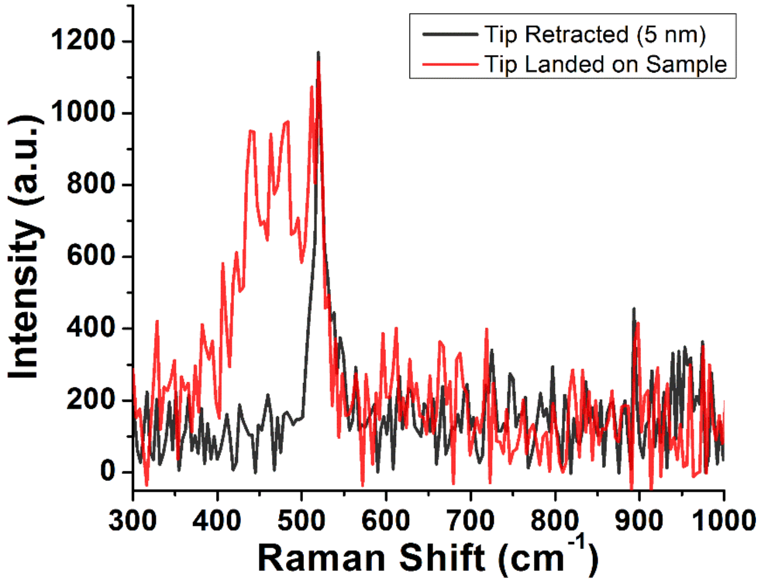

Figure S4. The "tip-in and tip-out" test for TERS enhancement factor evaluation.

It was found that even when the tip was retracted by 5 nm from the a-Si surface, the 1st order TO mode intensity at ~473 cm^{-1} of a-Si is significantly reduced (Figure S4), demonstrating that the near-field in the vicinity of the tip contributes most to the TERS signal. The near-field intensity (I_{near}) of the TO mode is estimated to be 850. With tip retracted from the a-Si surface by 5 nm, the intensity of the TO mode reduced to approximately 100 (i.e. far field intensity, I_{far} =100). The laser spot size is estimated to be 1 µm with the penetration depth of roughly 20 nm. The far-field sample volume (V_f) is thus estimated at 0.016 $µm^3$. The illuminating spot of the underneath the tip as shown in Figure S1 has an estimated radius

of 5 nm. Assuming the same penetration depth in a-Si as the far-field, the near-field scattering volume (V_n) is then estimated to be 1.57 x 10^{-6} μm^3. The enhancement factor, EF can then be calculated as, [1]

$$EF = \frac{I_{near}}{I_{far}} \cdot \frac{V_{far}}{V_{near}} \tag{1}$$

The calculated EF in this study is ~8 × 10^4.

Overview of all TERS spectra on the scanned area

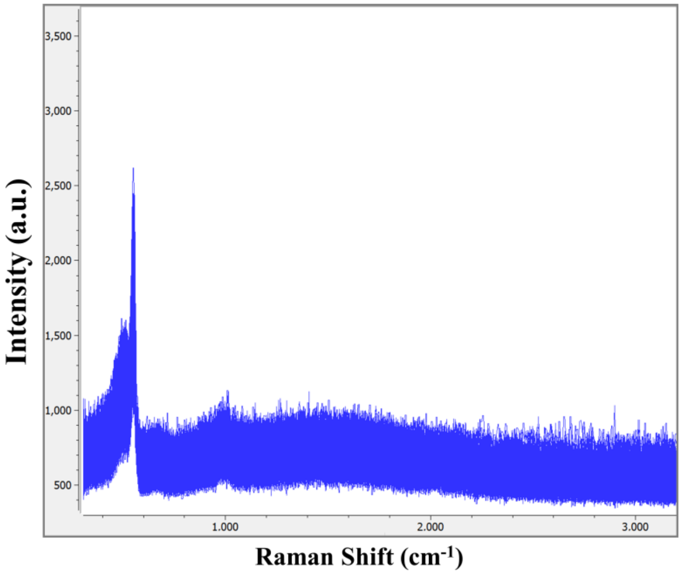

Figure S5. Overview of the TERS spectra collected from the scanned area on a-Si.

To explore the possible TERS enhancement of the 3rd and 4th order phonon modes of the a-Si, we went through all TERS spectra in the scanned area. Using 473 cm^{-1} as the peak center for 1TO mode for a-Si, there is no noticeable TERS peaks showing up at around ~1419 cm^{-1} (3TO) and 1892 cm^{-1} (4TO) shown in Figure S5. To more quantitatively illustrate this point, we resorted to the DFT calculation shown in Figure 6. Assuming that TERS has the same relative intensity of the fundamental 1TO mode versus higher phonon modes with that for INS, the intensity of the 3TO is 15.4% of 1TO, and that of the 4TO mode is 6.1%. The average signal-to-noise ratio is estimated to be 15%. Therefore, the reason that the 3TO and 4TO phonon modes are lacking is probably due to the unfavorable signal-to-noise ratio under the current experimental conditions.

Spatial resolution estimate

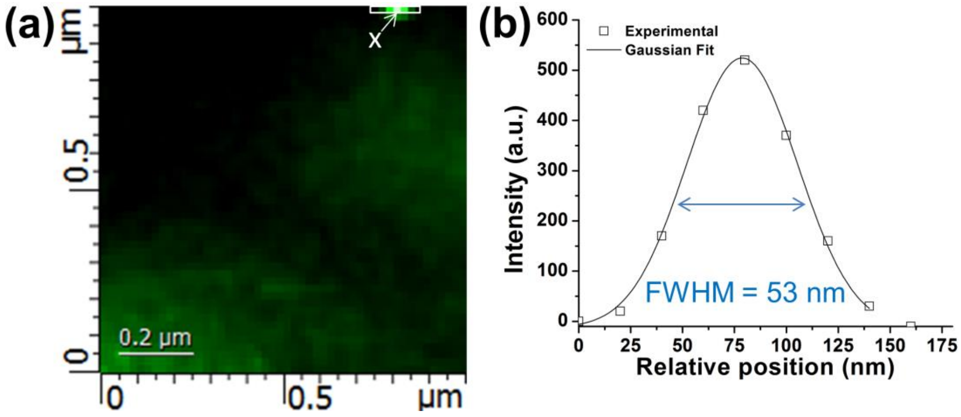

Figure S6. (a) TERS mapping of the single band centered at 2435 cm^{-1} (X-mode of the O_x-Si-H_y). (b) The intensity of the 2435 cm^{-1} peak across the spots marked in the white rectangle in (a), with "x" representing the center spot (maximum intensity).

To evaluate the spatial resolution of TERS in our experiments conducted on a-Si, line profiles of the x-mode band intensity along the spots marked by the rectangle in Figure S6(a) is fitted by the Gaussian function (Figure S6(b)). The Gaussian fit has a full width at half maximum (FWHM) of 53 nm, which is slightly larger than the tip diameter (42 nm), and more than twice that of the scanning step size. The method used to evaluate the TERS lateral spatial resolution agrees with other reports. [2, 3]

Evolution of manifold layout against different iterations

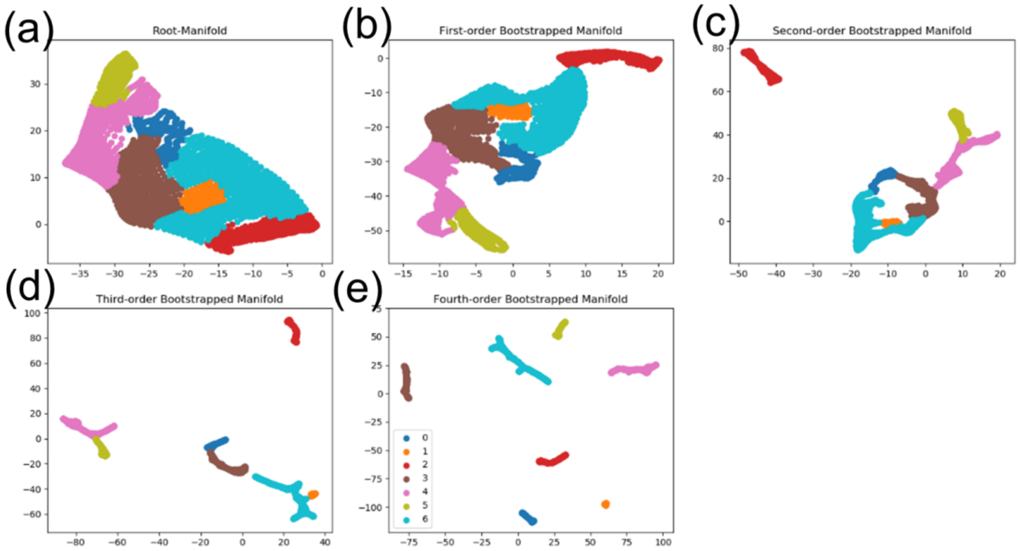

Figure S7. Manifold layouts overlaid with same set of cluster labels in Figure 4(a), (a) root manifold and (b,c,d,e) 1st, 2nd, 3rd and 4th-order manifold layouts during iterations of graph-bootstrapping procedure.

Topographic artifact estimate

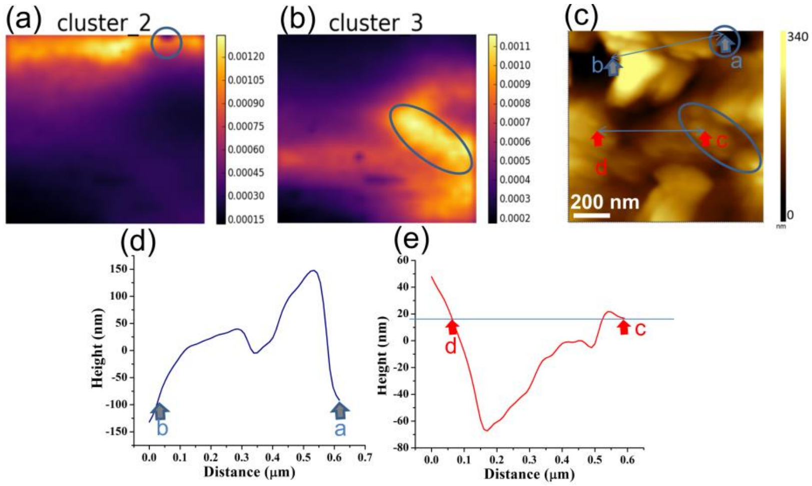

Figure S8. (a-b) Similarity loading of Clusters 2 and 3. (c) AFM height image of the scanned area. Arrows mark the crossline cut through which height was recorded, with arrow end pointing to two spots of the equal height. (d-e) The relative height profile marked by the two corresponding arrows in (c).

Upon analysis on several spots on the TERS mapping and the corresponding AFM height image, we failed to find a direct correlation between the a-Si surface TERS and the topography. Shown in Figure S8 (a), the black singular point presenting marked by the circle has a relative height of -99 nm as shown in Figure S8(c) marked by Point a. Point b along the Arrow ab in Figure S8(c) has an equal relative height of Point a. As discussed in Figure 5, Point a presents the X-mode in TERS spectrum, whereas Point b does not.   Another example is shown in Figure S8(b), where the ellipse marks Cluster 3 TERS spectra, in which Point c has the same height of Point d in Figures S8(c) and (e). However, the TERS spectrum taken from Point d falls into Cluster 6 (Figure 4). Therefore, it is manifest that TERS spectra taken from the sampling points of the same height in the AFM topography do not necessarily bear the same similarity.

At this stage, we cannot rule out the topographic artifacts that affect the TERS mapping due to the intrinsic technological limitations related to AFM and TERS. AFM images are taken due to the physical interaction between the scanning tip and the sample surface through piezoelectric ceramic scanners (definition). Any factors affecting this interaction influence the resultant AFM images. These factors include ai) geometrical shape and size of the tip; aii) hysteretic behavior of the piezoelectric scanner; aiii) thermal drift of the sample etc. [4] Regarding Factor ai, the tip has an end radius of ~20 nm in our current system. Therefore, any feature smaller than 20 nm would be convoluted on lateral adjacent points. However, the FDTD simulation (Figure S1) clearly shows that the size of the hot spot underneath the tip is on the scale of 10 nm, thereby providing a TERS map with the lateral resolution surpassing the resolution of the AFM image. Factor aii originates from the fact that that the same driving signal of the piezoelectric scanner does not correspond to the same position when scanning back and forth. It leads to a slight lateral shift of a step-like feature on AFM image. Factor aiii is less significant in the current study as we lowered the Raman laser power to 25 µW and minimized the laser irradiation time to mitigate the laser induced thermal effect. In addition to the intrinsic AFM topological artifacts, surface roughness could affect the TERS mapping due to the following reasons, bi) tip-sample distance varies during the tip raster across the sample plane to affect the TERS intensity of the a-Si; bii) nanoscale roughness may modulate the TERS signal intensity by up to 10 folds. [5] All above-mentioned factors lead to the mismatch of the TERS mapping (i.e. intensity of a vibrational mode) and the sample topography.

However, we point out here that the topographical artifact does not alter the major conclusion in the current study. The clustering algorithm applied is not dependent on the intensity of one or two TERS bands, but rather on the overall similarity of the TERS spectra within the scanned area. This is evident from Figure 3(d), in which the a-Si TO mode varies in the peak center, which is related to the local Si-Si bond and length distortions. The topographical artifact influencing the TERS peak shift of amorphous silicon is a subject worth exploring in future. To accommodate such a study, one will need well-controlled geometric factors for both the tip and the sample. A better model system should include a sharper TERS tip, and a smoother a-Si thin film coated on an atomic smooth substrate with reduced piezoelectric scanner hysteresis.

Evaluation of the high grating number on spectral resolution

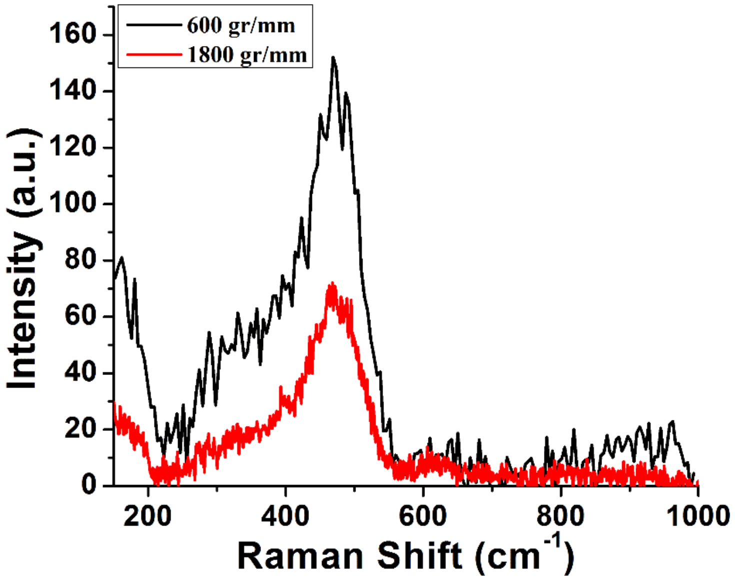

Figure S9. Comparison of the Raman spectra taken from a-Si using a high grating number (1800 gr/mm) and a low grating number (600 gr/mm), with improved laser power (600 µW).

The use of high-resolution grating can usually improve the spectral resolution. We further explored the possibility to physically deconvolute the overlapped Raman modes of the a-Si by using a grating with 1800 gr/mm. To avoid the interference of the 520 cm^{-1} peak from c-Si, only a-Si thin film was focused on by a standard confocal Raman microscopy. However, there was no noticeable improvement of the Raman spectral resolution compared to that collected by a 600 gr/mm grating, as seen from Figure S9.

Possible reaction paths of the water with the a-Si

It has been reported previously that silicon nanometer objectives reacted with the oxidation agent, water to generate hydrogen following the reaction path [6, 7]

$$Si + 2H_2O \rightarrow SiO_2 + 2H_2 \uparrow$$

Thus, it is reasonable that the surface unsaturated Si bonds for as sputtered a-Si are capable of splitting water. We herein propose intermittent reaction between water and a-Si as

$$2Si + H_2O \rightarrow SiOH + SiH$$

This reaction path explains the vibrational modes based on Si-OH and Si-H moieties observed in INS spectrum in Figure 6. The existence of the Si-OH from water reaction with a-Si agrees with what was reported by Liao, et al. [8] It should be noted that we cannot rule out the existence of the Si-O-Si in the current sample. Therefore, the surface chemistry of the a-Si may include Si-O-Si, Si-OH, Si-H and unreacted Si-Si groups.

Neutron PDF profiles of a-Si and c-Si

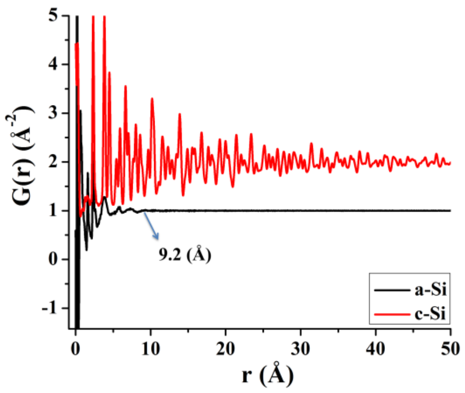

Figure S10. Comparison of the neutron PDF data between the amorphous silicon (a-Si) used in the current study and a crystal silicon (c-Si) standard sample.

Neutron PDF plot was used to estimate the phonon coherence length of the a-Si thin film in the current study. As shown in Figure S10, in contrast to crystalline silicon, a-Si does not possess long-range translational order, as manifested by the fact that its pair distribution function (G(**r**)) does not show noticeable peak at above 9.2 Å. G(**r**) correlates to the neutron powder diffraction data through the Fourier transform of the scattering structure function S(**Q**) by

$$G(\boldsymbol{r}) = \frac{2}{\pi}\int_0^\infty Q[S(\boldsymbol{Q}) - 1]\sin(\boldsymbol{Qr})d\boldsymbol{Q} \qquad (2)$$

The TERS lateral light confinement width, L_w is estimated by ~ $\sqrt{2hr_o}$, [9] in which h is the tip-sample distance, h = 2 nm and r_o is the curvature radius of the tip apex and estimated to be 21 nm). L_w is thus estimated to be 9.17 nm, agreeing fairly well with that estimated from FDTD simulation as shown in Figure S1. Note that the coherent TERS response can be observed only if the excitation spot size is

comparable to or smaller than the phonon coherence length, [9] we thus reason that the incoherent scattering is the major contribution to the TERS spectra.

Surface free energy evaluation for a-Si thin film sample

As noted by Saito et al. in an early study, the surface free energy difference between the a-Si and c-Si mainly stems from the bond angle distortions. [10] The strain energy per mole of the a-Si can be related to the bond angle distortions by

$$U_{\Delta\theta} = N_A \cdot 6 \cdot [\frac{1}{2} \cdot k_\theta \cdot (r \cdot \Delta\theta)^2] \cdot 0.0003046 , \quad (3)$$

where k_θ is the force constant for the Si-Si bond bending; r is the estimated Si-Si distance (~2.35 A); the number of bond pairs per each Si atom is 6 and the summation is over all Si embedded in the random a-Si network, NA is the Avogadro's constant. The factor of 0.0003046 is used to convert the energy unit to 'Joule'. k_θ is related to the Keating potential force constant, β using $k_\theta = 2/3\beta$. The value of β can be estimated over a range of 6.7-9.7 N/m, [10, 11, 12, 13] leading to k_θ over a range of 4.5-6.5 N/m. Taking the minimum value of k_θ here, the estimated surface free energy is calculated to be 23 KJ/mole, nearly twice of which corresponding to the minimum distortion angel (6.6^o) at 12 KJ/mole. This indicates that the surface amorphous Si on the thin film surface used is highly disordered and far from the equilibrium structure in the current study

The stability ratio calculation for SiO_z based on effective media theory

For the case of the SiO_z ($0 < z < 2$) as the next-nearest neighbors, a geometric average SRX is given by [14, 15]

$$X_{SiOz} = [X_{Si} X_O^z]^{1/(1+z)} \quad (4)$$

For z= 2, the SRX electronegativity, X_{SiOz} can be calculated as 4.14.

Supporting Information Reference

1. Mehtani D*, et al.* Nano‐Raman spectroscopy with side‐illumination optics. *Journal of Raman Spectroscopy: An International Journal for Original Work in all Aspects of Raman Spectroscopy, Including Higher Order Processes, and also Brillouin and Rayleigh Scattering* **36**, 1068-1075 (2005).

2. He Z*, et al.* Tip-enhanced Raman imaging of single-stranded DNA with single base resolution. *Journal of the American Chemical Society* **141**, 753-757 (2018).

3. Nanda J*, et al.* Unraveling the nanoscale heterogeneity of solid electrolyte interphase using tip-enhanced Raman spectroscopy. *Joule* **3**, 2001-2019 (2019).

4. Ricci D, Braga PC. Recognizing and avoiding artifacts in AFM imaging. In: *Atomic Force Microscopy*). Springer (2004).

5. Zhang W, Cui X, Yeo B-S, Schmid T, Hafner C, Zenobi R. Nanoscale roughness on metal surfaces can increase tip-enhanced Raman scattering by an order of magnitude. *Nano letters* **7**, 1401-1405 (2007).

6. Erogbogbo F*, et al.* On-demand hydrogen generation using nanosilicon: splitting water without light, heat, or electricity. *Nano letters* **13**, 451-456 (2013).

7. Goller B, Kovalev D, Sreseli O. Nanosilicon in water as a source of hydrogen: size and pH matter. *Nanotechnology* **22**, 305402 (2011).

8. Liao WS, Lee SC. Water-induced room-temperature oxidation of Si–H and–Si–Si–bonds in silicon oxide. *Journal of applied physics* **80**, 1171-1176 (1996).

9. Kharintsev SS, Saparina SV, Fishman AI, Stolov AA, Li J. Spectrally Resolving Coherent TERS Spectroscopy of Electrically Biased Carbon-Coated Fibers. *The Journal of Physical Chemistry C*, (2020).

10. Saito T, Karasawa T, Ohdomari I. Distortion energy distributions in the random network model of amorphous silicon. *Journal of Non-Crystalline Solids* **50**, 271-276 (1982).

11. Alben R, Smith Jr J, Brodsky M, Weaire D. Theory of infrared and Raman spectra of amorphous Si and Ge. *Physical Review Letters* **30**, 1141 (1973).

12. Alben R, Weaire D, Smith Jr J, Brodsky M. Vibrational properties of amorphous Si and Ge. *Physical Review B* **11**, 2271 (1975).

13. Biswas R, Bouchard A, Kamitakahara W, Grest G, Soukoulis C. Vibrational localization in amorphous silicon. *Physical review letters* **60**, 2280 (1988).

14. Carver JC, Gray RC, Hercules DM. Remote inductive effects evaluated by x-ray photoelectron spectroscopy (ESCA). *Journal of the American Chemical Society* **96**, 6851-6856 (1974).

15. Tsu D, Lucovsky G, Davidson B. Effects of the nearest neighbors and the alloy matrix on SiH stretching vibrations in the amorphous SiO r: H (0< r< 2) alloy system. *Physical Review B* **40**, 1795 (1989).
